# Morphometric Characteristics of Rat Testes Tissue After Exposure to Dust–Salt Aerosols of the Aral Sea

**DOI:** 10.3390/biology14040380

**Published:** 2025-04-07

**Authors:** Assylgul Suleimen, Bibigul Rakhimova, Svetlana Jangildinova, Aidar Aitkulov, Bayan Yessilbayeva, Karlygash Yestemessova, Bayan Dyussenbekova, Khamida Abdikadirova, Gulzhazira Turlybekova, Kymbat Amreyeva

**Affiliations:** 1 Department of Biomedicine, Karaganda Medical University, Karaganda 100012, Kazakhstan; suleymena@qmu.kz (A.S.); dzhangildinova@qmu.kz (S.J.); esilbaeva@qmu.kz (B.Y.); estemesova@qmu.kz (K.Y.); dyusenbekova@qmu.kz (B.D.); 2 Department of Physiology, Buketov Karaganda University, Karaganda 100024, Kazakhstan; aitkulov_aidar@buketov.edu.kz; 3 Department of Physiology, Karaganda Medical University, Karaganda 100012, Kazakhstan; abdikadirova@qmu.kz; 4 Department of Zoology, Karaganda University Named After E.A. Buketov, Karaganda 100024, Kazakhstan; gulzhazira_t@mail.ru; 5 School of Public Health, Karaganda Medical University, Karaganda 100012, Kazakhstan; amreeva@qmu.kz

**Keywords:** Aral Sea, dust–salt aerosols, rat, testes, morphometry

## Abstract

The long-term reduction of the Aral Sea area has led to an ecological catastrophe and a deterioration in the health of the population. The progressing demographic crisis necessitates research on factors that negatively impact the fertility of the population in the Aral region. To investigate the influence of Aral Sea dust–salt aerosols on rat testicular tissues, we exposed male rats to the inhalation of dust–salt aerosols for 7, 24, 30, 48 and 72 days. The results of the morphometric analysis of the rat testes tissues indicate that aerosols containing salts from the Aral Sea have a detrimental effect on the seminiferous epithelium, causing a depletion of the spermatogenic layer and reduction of seminiferous tubules lumen. The morphological signs of testicular damage in the experimental animal groups included low cellularity of seminiferous tubules, vacuolization of Sertoli cells, and a decrease in the number of Leydig cells in the interstitium. The smallest thickness of the spermatogenic layer and the lowest number of Leydig cells were observed in rats after the longest inhalation exposure to the dust–salt aerosols.

## Simple Summary

The significant shrinkage of the Aral Sea, located in Central Asia, is one of the major anthropogenic environmental disasters of recent decades. The exposure of the seabed has led to the formation of the Aralkum Desert, created by human activities. This artificial desert is a source of frequent and prolonged dust–salt storms in the surrounding regions. In our experiment, we simulated the conditions of a dust–salt storm to determine how this natural phenomenon affects the structure of the testes in laboratory animals. We found that inhalation exposure to dust–salt aerosols leads to a decrease in the thickness and diameter of the seminiferous tubules, a reduction in the number of hormone-producing and supporting cells, and disruptions in germ cell differentiation in the testes of rats. It is possible that similar disruptions may also occur in men living in areas affected by dust storms, negatively impacting their reproductive health. Therefore, the findings of this study can be used to develop measures aimed at improving the demographic situation in the Aral Sea region of Kazakhstan.

## 1. Introduction

The loss of the Aral Sea is a striking example of how disturbance of the natural landscape contributes to dust and health impacts. Intensive water diversion upriver for farming has exposed the seafloor and salty flats, which are now the sources of severe dust storms [1]. The severely impacted zone was mainly distributed around the Aral Sea region, including the western part of Kazakhstan and most of Uzbekistan and Turkmenistan, with an area of approximately 2 million km^2^ [2].

The millions of people living in this region are suffering not only from natural disasters but also from a sharp decline in their health status. Children living in the region of the Aral Sea suffer from cerebral–endocrine pathologies, hereditary diseases, somatogenic diseases, constitutional growth delay, a toxic chemical load, and ecologically determined short stature [3]. Among congenital developmental defects in children in this region, cardiovascular defects, defects of the nervous system, and multiple defects prevail. These defects can be considered as indicators of ecological distress in the region [4]. Men and women residing in close proximity to the Aral Sea experience fertility problems [5,6,7]. It was also found that cancer morbidity in the adult population residing in the Aral Sea region is 1.5 times higher than in environmentally favorable regions of Kazakhstan [8].

A possible cause of many diseases of the reproductive sphere and other systems of organs is the strong oxidative stress observed in men and women living near the shrinking Aral Sea [9,10].

Given the progressing demographic crisis in Kazakhstan, research on the reproductive status of the population in environmentally adverse areas, such as the Aral Sea region, and the identification of factors that negatively affect fertility become particularly relevant. One of the key factors leading to infertility in couples is the disruption of spermatogenesis.

Directly investigating the influence of Aral Sea dust–salt aerosols on the reproductive system is challenging because many different factors influence the fertility of men and women in environmentally adverse areas. However, identifying the nature of reproductive function disruptions in the population under the influence of dust–salt aerosols, which are the primary environmental hazard in the Aral region, may allow the development of methods for preventing and treating reproductive system diseases in the region.

In connection with this, the aim of this study was to identify changes in the morphometric characteristics of rat testicular tissues after the experimental dusting of adult male rats with dust–salt aerosols from the Aral Sea. This investigation was conducted as a continuation of the research [11].

## 2. Materials and Methods

### 2.1. Research Object

The objects of the experimental study were 42 adult (four-month-old) male white laboratory Wistar rats (vivarium of Karaganda Medical University, Kazakhstan) weighing 180–220 g, with 35 rats in the experimental groups and 7 rats in the control group. The animals in the experimental group were divided into 5 groups: I—with inhalation exposure for 7 days, II—with exposure for 24 days, III—with exposure for 30 days, IV—with exposure for 48 days, V—with exposure for 72 days.

The animals were housed under standard conditions in the vivarium (23 ± 1 °C, 60–70% humidity and a 12/12 light/dark cycle) and supplied with nutritionally adequate food according to standards of the European Convention for the Protection of Animals Used for Scientific Purposes [12]. The study design was reviewed and approved by the ethics committee of Karaganda Medical University (Approval Code: protocol no. 6, Approval Date: 6 October 2017).

### 2.2. Inhalation Exposure

Inhalation exposure was conducted on male white laboratory rats in a specialized inhalation chamber with a cylindrical shape and a volume of 150 L. The animals were placed inside the chamber for 4 h a day following the protocol of [13]. For the exposure, a dust–salt mixture from the bottom of the Aral Sea was used, previously ground into fine aerosol particles with sizes up to 5 μm. The calculation of the dosage of pre-Aral Sea salt was based on sodium chloride, as its content in the dust samples from the Aral Sea, according to preliminary tests, was the highest compared to other salts. Rats in the experimental groups were exposed to dust–salt aerosols at a concentration of 1.5 mg/m^3^, which is considered toxic according to hygiene criteria for atmospheric air approved in Kazakhstan [14]. This concentration adequately reflects the conditions in the areas adjacent to the Aral Sea based on data on the average monthly amount of deposited aerosols [1] and their distribution in the atmosphere [15]. The composition and quantitative content of minor components in Aral Sea dust are presented in our article [16]. The chemical composition of the aerosol was analyzed using atomic absorption spectrometry, identifying 30 minor components. The highest mass fractions were found for silver, titanium, strontium, manganese, and barium (listed in descending order).

### 2.3. Ethical Permission

At the end of the experiment, the rats from the control and experimental groups were removed from the experiment using the method of partial decapitation under light ether anesthesia, following the rules for working with laboratory animals according to the “International Recommendation for Conducting Medical and Biological Research Using Animals” [17].

### 2.4. Fixation and Staining Methods

The testes were fixed in Bouin’s fixative (saturated picric acid (Sisco Research Laboratories, Mumbai, India)—75 mL, formalin (Scharlab, Barcelona, Spain)—25 mL, glacial acetic acid (Thermo Scientific Chemicals, Waltham, MA, USA)—5 mL). To exclude the influence of anatomical features of blood supply on the results, the right testis of the rats was selected for morphological analysis.

Tissue samples were dehydrated in 5 changes of isopropanol (Sigma-Aldrich, Burlington, MA, USA) in a tissue processor, cleared and infiltrated in 2 portions of a mixture of mineral oil (Sisco Research Laboratories, India) and isopropanol, then in pure mineral oil, and finally impregnated with paraffin (3 changes) at 58 °C. Blocks were prepared using the Sakura Tissue-Tek TEC-5 embedding station (Sakura Finetek, Tokyo, Japan). Sections were cut using the Sakura Accu-Cut^®^ SRM™ 200 rotary microtome (Sakura Finetek, Tokyo, Japan), with a thickness of 4–6 µm. The slides were stained with hematoxylin-eosin (Thermo Scientific Chemicals, USA), and control tests were performed for histochemical reactions. The stained slides were mounted with a synthetic medium called “Histomount” (Thermo Scientific Chemicals, USA) under a coverslip.

The stained slides for studying the microstructure of the testes were examined under a Nikon Eclipse Ci light microscope (Nikon Corporation, Tokyo, Japan) at magnifications of ×40, ×100, ×200, and ×400. The sections were photographed using a DS-Fi2 camera (Nikon Corporation, Tokyo, Japan).

### 2.5. Morphometric Parameter Assessment

The following morphometric parameters of the testicular tissue were evaluated: the diameter of coiled seminiferous tubules in cross-section, the thickness of the germinal layer (spermatogenic layer), the number and vacuolization of Sertoli cells, and the number of Leydig cells [18]. The number of Sertoli cells was counted under a light microscope and then divided by the number of counted seminiferous tubules. The number of Sertoli cells showing signs of intracellular microvacuolization or swelling, affecting the basal region of Sertoli cell cytoplasm and causing displacement and disorganization of spermatogenic cells, was also counted. Quantitative and qualitative indicators of positively stained spermatogenic, somatic, and endocrine cells were evaluated in the light microscope field of view (magnifications of ×100 and ×200) in 10–40 restricted fields of view.

A minimum of 50 histological slides were examined for each experimental group. The counting of germ cells and Sertoli cells in seminiferous tubules was performed on 10 strictly transverse sections of seminiferous tubules per sample.

The testis index was determined as the percentage ratio of testis weight (right and left) to the total body weight of the animal.

### 2.6. Statistical Analysis

For the morphological evaluation of testicular tissue, the S.G. Johnsen scale was used (a scale for assessing the degree of spermatogenesis depletion from 1 to 10 points) [19].

Statistical analysis of the results was performed using the “Statistica” software package, version 8.0. The median (Me) was used as a measure of central tendency, and the interquartile range (Q1–Q3) was used as a measure of dispersion. Differences between groups were analyzed using the non-parametric Kruskal–Wallis test (at the level of significance 0.05). Differences were considered statistically significant at *p* < 0.05.

## 3. Results

The testes of the rats in the control group were characterized by a normal histological structure corresponding to the identified stage of the spermatogenic cycle. In all histological sections, there was a sequential arrangement of spermatogenic layers, spermatozoa, and interstitial tissue with Leydig cells.

**Figure 1:**
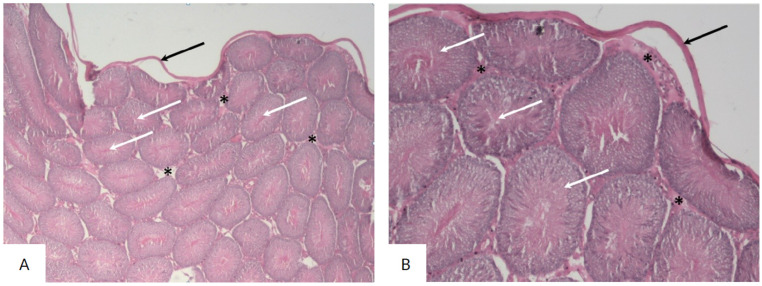
Microphotograph of the testes of rats in the control group. The testis is covered by the “tunica albuginea” (black arrow), and it contains seminiferous tubules (white arrow), which appear predominantly round, equal in size and shape, lined with orderly rows of spermatogenic cells at different stages of maturation. The interstitium (asterisk) contains thin loose Leydig cells (hematoxylin and eosin, ×40 (**A**), ×100 (**B**)).

The testes were covered with a capsule made of connective tissue (tunica albuginea). The testicular parenchyma consisted of round and oval-shaped seminiferous tubules with smooth and regular contours. The seminiferous tubules were uniform in size and shape and were lined with regularly arranged rows of seminiferous ducts, composed of spermatogenic cells at different stages of maturation. The epithelium was formed by normal spermatogenic layers, represented by spermatogonia, primary and secondary spermatocytes, spermatids, and spermatozoa. The interstitial spaces between the tubules contained Leydig cells (Figure 1).

**Figure 2:**
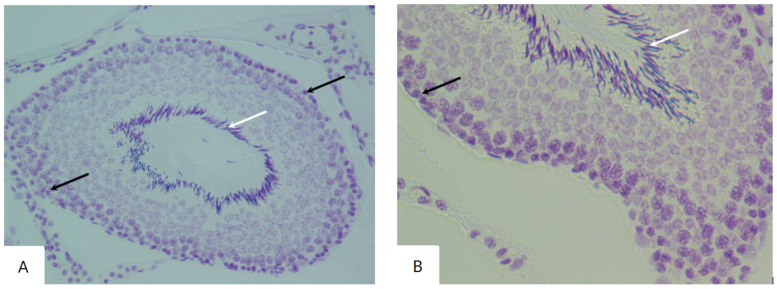
Sections of the testes of rats in the control group stained with the Feulgen reaction. Spermatogenic cells (black arrow) and spermatozoa (white arrow) (Feulgen reaction, Carnoy’s fixative, ×200 (**A**), ×400 (**B**)).

The use of the Feulgen reaction in the control group revealed a large number of spermatozoa in the lumen of the tubules and a uniform staining of the spermatogenic cells (Figure 2).

Thus, the testes of rats in the control group demonstrated a normal histological structure with active, mature functioning seminiferous tubules, characterized by a complete spermatogenic cycle.

**Figure 3:**
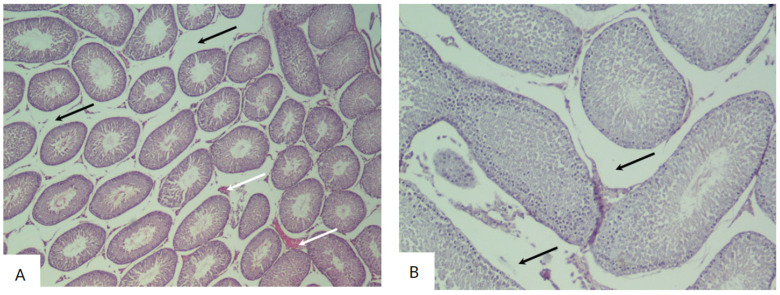
Sections of the testes of rats in the I experimental group (exposed for 7 days): (**A**) Diffuse damage characterized by interstitial edema (black arrow) with congested blood vessels (white arrow) (hematoxylin and eosin, ×40). (**B**) Increased interstitial space with changes in the number of Leydig cells (hematoxylin and eosin, ×100).

**Figure 4:**
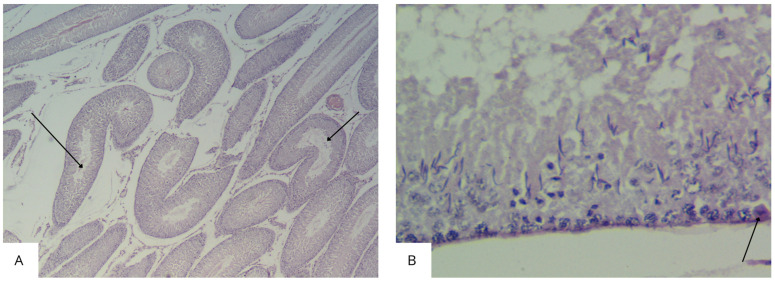
Sections of the testes of rats in experimental group II (exposed for 24 days): (**A**) The testis contains seminiferous tubules (black arrow), which appear uneven in size and shape (hematoxylin and eosin, ×40). (**B**) Irregular contour of the seminiferous tubule, degeneration of the spermatogenic epithelial layer, and presence of apoptotic cells (black arrow) (hematoxylin and eosin, ×400).

The histological examination of the testicular sections of rats in the experimental groups I–V demonstrated the preservation of the morphological pattern with well-defined sections of the seminiferous tubules. In certain fields of view, irregular outlines of the tubules were observed, which had different shapes and sizes. These groups also showed a significant number of degenerating spermatogenic cells. The interstitium exhibited pronounced edema with congested blood vessels (Figure 3 and Figure 4).

The Feulgen reaction in the testicular tissues of the experimental groups revealed a large number of spermatozoa in the lumen of the tubules and uneven staining of the spermatogenic cells.

In experimental group I, there was a lower number of spermatogonia compared to the control group. Spermatocytes acquired an oval, less commonly spherical, shape. In some tubules, late spermatids were indistinguishable. Coiled tubules were observed in which spermatozoa were absent from the lumen.

In the tubules of rats in experimental groups II and III, spermatogenic cells with dark round nuclei were observed near the basal compartment. In some tubules, large multinucleated cells were detected in the spermatogenic epithelium near the lumen. In the altered tubules, the nuclei of Sertoli cells had an irregular shape and heavily stained chromatin. Disorganized round and elongated spermatids were found unevenly distributed in the basal compartment and intraluminal spaces, indicating the loss of spermatogenic cells.

**Figure 5:**
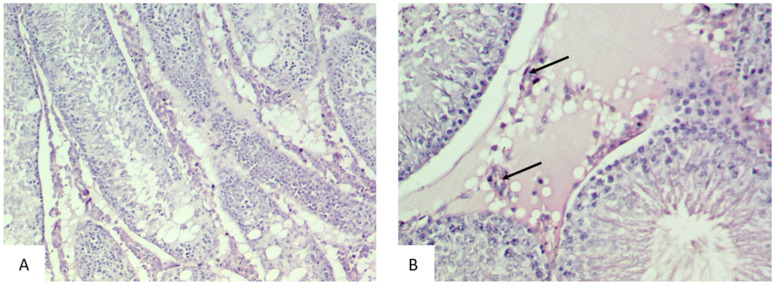
Sections of the testes of rats in experimental group III (exposed for 30 days): (**A**) The testis contains seminiferous tubules with signs of microvacuolization, affecting the basal region of Sertoli cell cytoplasm and causing displacement and disorganization of spermatogenic cells (hematoxylin and eosin, ×100). (**B**) Leydig cells with hyperchromatic nuclei (black arrow) (hematoxylin and eosin, ×400).

In the tissue sections of the testes from experimental group III, Leydig cells with hyperchromatic nuclei were observed in the interstitium (Figure 5).

**Figure 6:**
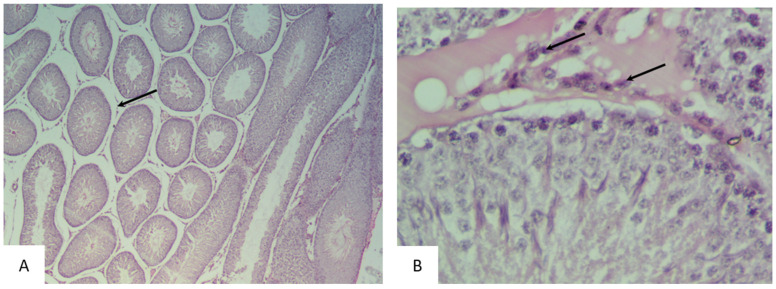
Sections of the testes of rats in the IV experimental group (exposed for 48 days): (**A**) The testis contains seminiferous tubules, which appear uneven in size and shape. The stroma exhibits pronounced edema (black arrow) (hematoxylin and eosin, ×40). (**B**) Leydig cells with prominently stained vesicular nuclei (black arrow) (hematoxylin and eosin, ×400).

The histological examination of the testicular sections of rats in the experimental group IV (considering the identified stage of the spermatogenic cycle) demonstrated a significant reduction in the number of spermatozoa in the lumen of some tubules. Basal cells with hyperchromatic proliferating nuclei were also observed. The basal membrane was preserved in the majority of the sections, but its integrity was disrupted in some tubules. In some sections, the basal membrane was thickened and heterogeneous. Leydig cells had normal stained nuclei (Figure 6).

**Figure 7:**
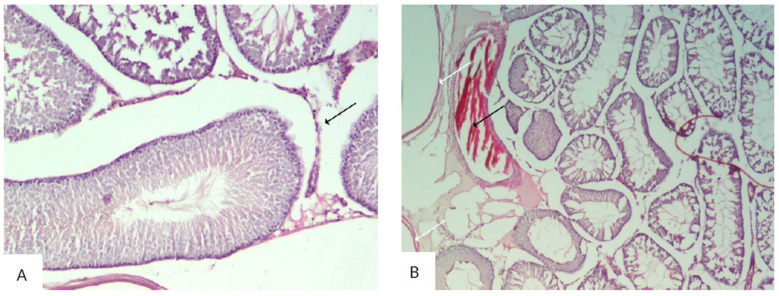
Sections of the testes of rats in experimental group V (exposed for 72 days): (**A**) Diffuse damage characterized by interstitial edema (black arrow) with pronounced congestion of blood vessels (hematoxylin and eosin, ×100). (**B**) Increased interstitial space with a decrease in the number of Leydig cells, thickening of the capsule (white arrow), and pronounced congestion of blood vessels (black arrow) (hematoxylin and eosin, ×40).

In rats from experimental group V, desquamated damaged epithelial cells were observed in the lumen of some tubules (less than 20% of the tubules). Vacuoles appeared between the damaged cells, replacing them. A large number of spermatozoa were observed in the lumen of some tubules. Some seminiferous tubules (less than 3%) were ruptured, and the basal membrane was damaged. Interstitial expansion with the appearance of vacuoles and congestion of blood vessels was also noted (Figure 7).

The conducted morphometric study showed that in rats from experimental groups I–III, the median diameter of seminiferous tubules significantly decreased compared to the control (Table 1). Throughout the experiment, the thickness of the spermatogenic layer decreased in rats from the experimental groups (except the IV group). In experimental group V, the thickness of the spermatogenic layer decreased by 22% compared to the control group. Exposure to dust and salt aerosols led to a significant decrease in the number of Sertoli cells in the testicular tissues of rats in the I, IV and V experimental groups, ranging from 1.6 to 1.9 times lower than the control. Furthermore, the vacuolization of Sertoli cells was more frequently observed in the experimental groups. The number of Leydig cells decreased in rats from the IV and V experimental groups, depending on the duration of exposure to dust and salt aerosols. The lowest number of Leydig cells (59% of the control group) was observed in rats from experimental group V.

**Figure 8:**
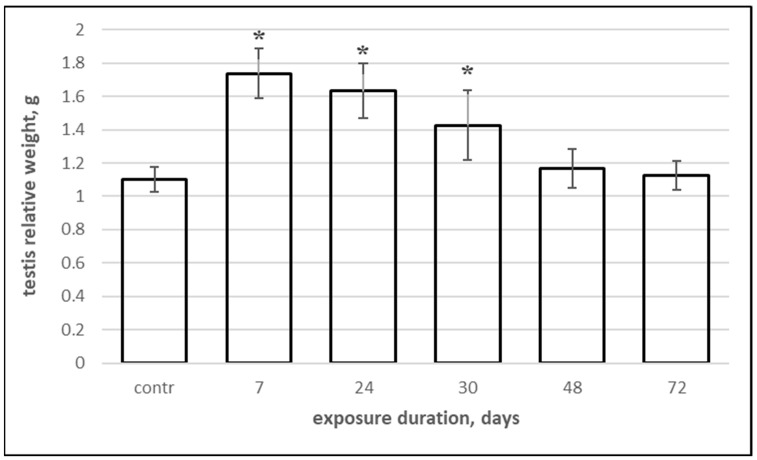
Testis index depending on the duration of inhalation exposure to Aral Sea dust–salt aerosols. *—statistically significant differences from the control group (*p* < 0.05).

A significant increase in the testis index was observed in rats exposed to dust–salt aerosols for 7, 24, and 30 days compared to the control group (Figure 8). Longer exposure did not have a similar effect on the relative weight of the testes.

**Figure 9:**
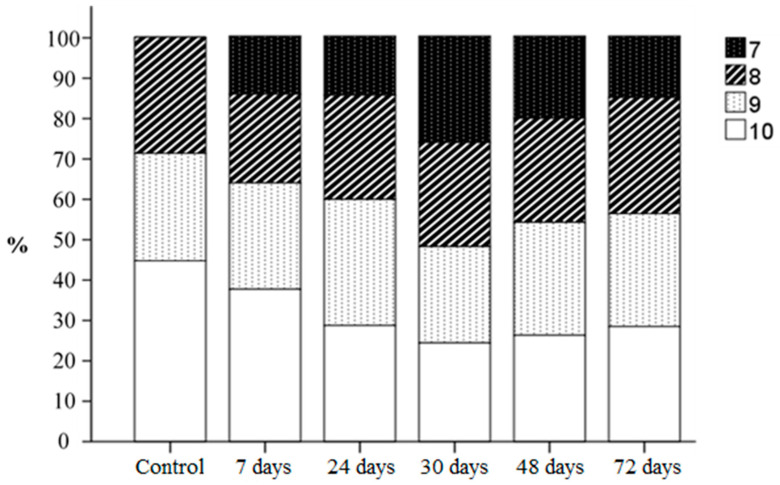
Histological examination of testicular biopsies (according to the Johnsen S.G. scale).

During histological examination of testicular specimens, it was found that normal spermatogenesis (scored 10 points according to the Johnsen S.G. scale) was observed in 45% of seminiferous tubules in the control group of rats and in 27–38% of tubules in the experimental groups (Figure 9).

**Figure 10:**
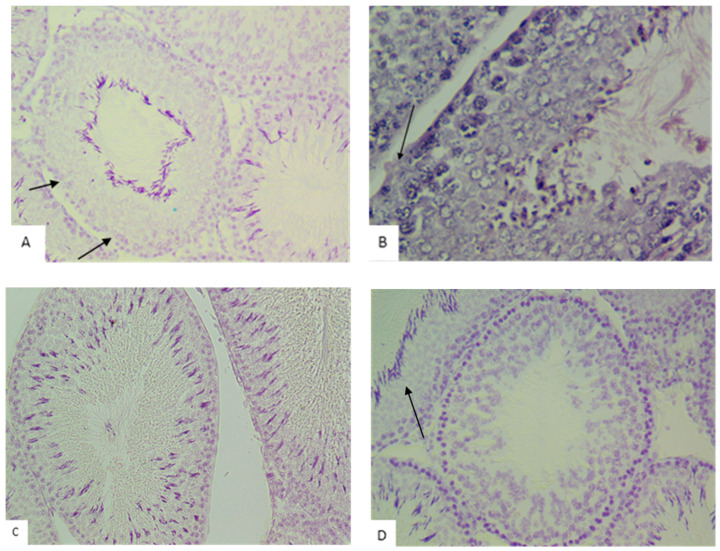
Microphotographs of rat testes with varying degrees of spermatogenesis disruption (according to the Johnsen S.G. scale): (**A**,**B**)—Mild disorganization of the cytoarchitecture of the seminiferous epithelium with partial loss of spermatogenic cells (black arrow), score: 9 (hematoxylin and eosin, ×400); (**C**)—Fewer than five spermatozoa and a reduced number of elongated spermatids, score: 8 (Feulgen reaction, ×400); (**D**)—Absence of spermatozoa and late (elongated) spermatids, score: 7; adjacent tubule with intact spermatogenesis (black arrow), score: 10 (Feulgen reaction, ×400).

Minor disturbances in spermatogenesis, such as disorganization of the spermatogenic epithelium and a high number of late spermatids (scored 9 points), were observed in 27% of tubules in the control group and in 23–32% of tubules in the experimental groups (Figure 10). Less than five spermatozoa per tubule and a few late spermatids (scored 8 points) were detected in 28% of tubules in the control group and in 15–25% of tubules in the experimental groups. The absence of spermatozoa and late spermatids and a high number of early spermatids (scored 7 points) were observed only in the tubules of rats in the experimental groups. The highest number of tubules with these characteristics was noted in the group exposed to dust for 30 days (25%). In the other experimental groups, the morphological pattern corresponding to a 7-point assessment according to the S.G. Johnsen scale was observed in 15–19% of tubules.

Thus, the final score according to the S.G. Johnsen scale (Table 1) was significantly lower in the testicular tissues of rats in the III, IV and V experimental groups compared to the control group.

## 4. Discussion

The toxic effects of Aral Sea dust–salt aerosols on ovarian tissue in mice (rats) have been investigated [20], but data on the toxicity induced by these aerosols on the male reproductive system are absent. In recent years, several studies have focused on evaluating the effects of pulmonary exposure to small aerosol particles on male reproductive organs. For instance, it was identified in [21] that inhaling cellulose nanocrystals leads to interstitial edema and dystrophic changes in seminiferous tubules. Male infertility in this study is associated with enhanced oxidative stress in testicular tissues. Similar changes in rat testes were also observed after prolonged inhalation of diesel exhaust, which contains various gases and carbonaceous particles [22]. Another study on the reproductive toxicity of air-suspended particles found that vanadium particles lead to necrosis and a reduction in the number of spermatogonia, spermatocytes, and Sertoli cells, as well as vacuolization of cells [23]. Volcanic emissions, containing fine solid particles in addition to gases, caused a significant reduction in the diameter of seminiferous tubules and a decrease in the number of sperms in experimental animals [24]. Exposure to ambient levels of urban particulate matter also decreased tubular diameter and sperm production in the testes of mice in experiments [25]. A decrease in the diameter of seminiferous tubules and the thickness of the spermatogenic epithelium, along with a reduction in the number of Sertoli cells, was observed after exposure to crack-cocaine particles [26]. Prolonged inhalation of tobacco smoke, which represents an aerosol, led to a decrease in the number of spermatids and a reduction in the diameter of seminiferous tubules [27].

According to the results of our study, in all groups of rats exposed to Aral Sea salt aerosols, there was a pronounced increase in interstitial fluid with edema and stasis in the testicular blood vessels. We believe that this damage reflects disturbances in hemodynamics and endothelial damage of the vessels. The persistence of severe edema for a prolonged period may indirectly lead to hypoxia in the seminiferous tubules due to increased separation between blood vessels and tubules. Edematous interstitial fluid may contain proteins or other factors or generate active oxygen species that directly suppress the differentiation of spermatogonia or induce apoptosis, exacerbating the severity of testicular damage in the experimental animal groups. Significant swelling of testicular tissues is the most probable cause of the statistically significant increase in the relative weight of the testes in response to short-term exposure to dust–salt aerosols. Prolonged exposure (exceeding 30 days) leads to progressive dystrophic changes in testicular tissues and a decrease in their weight.

Seminiferous tubules in the control group of rats were the widest, likely related to the secretion of fluid by Sertoli cells to facilitate spermiation, while the smallest diameter was observed in rats exposed to Aral Sea salt aerosols for 7, 24, and 30 days (Table 1). According to data presented in [28], this is attributed to a reduction in Sertoli cell fluid secretion and may represent an initial stage of impaired spermiation. We believe that the reduction in the overall diameter of seminiferous tubules occurs as a result of the depletion of spermatogenic cells and decreased secretion of tubular fluid. The fluid in the seminiferous tubules is secreted by Sertoli cells and maintains the size of the lumen, which varies depending on the stage of spermatogenesis. This is an androgen-dependent function of Sertoli cells that is influenced by altered testosterone secretion. Another important regulatory factor for fluid secretion is the presence of elongated spermatids. Therefore, with depletion or damage to these cells, fluid production and the size of the tubular lumen decrease.

Thus, the nature of the damage to rat testes induced by inhalation exposure to Aral Sea dust–salt aerosols is similar to that observed in chronic inhalation of other fine particles. The results of the morphometric study indicate that Aral Sea salt aerosols have a negative impact on the seminiferous epithelium, causing moderate tubular atrophy with a reduction in the thickness of the spermatogenic layer. In addition to quantitative changes in the components of the seminiferous epithelium, qualitative morphological changes were observed, such as low cellularity of seminiferous tubules, focal vacuolization of Sertoli cells, and a decrease in the number of Leydig cells in the interstitium.

Johnsen’s score is a reliable quantitative histological system that can be successfully used for assessing testicular histopathology in animals [29]. The decrease in Johnsen’s score in the experimental groups indicates a poorer spermatogenic status compared to the control group. Under normal conditions, the full process of mammalian spermatogenesis involves the transformation of progenitor spermatogonia into mature spermatozoa through stages of primary and secondary spermatocytes and round and elongating spermatids. The absence or reduced number of elongating spermatids in some seminiferous tubules of rats in the experimental groups suggests impairments in the final stages of spermatogenesis.

It is well known that gonads contain a high proportion of unsaturated lipids, making the reproductive system highly sensitive to oxidative stress and lipid peroxidation. Reactive oxygen species (ROS) induce DNA strand breaks, damage lipid and protein molecules, disrupt mitochondrial function and cytoskeletal structure, and ultimately trigger apoptosis of hormone-producing, supporting, and spermatogenic cells [30,31].

Minor components of the Aral Sea dust–salt aerosols include heavy metals, which can pass through the blood–testis barrier and cause testicular damage. Therefore, to explain the molecular mechanism of the impact of dust–salt aerosol components on testicular tissues, it seems appropriate to further study the accumulation of heavy metals in the reproductive organs of experimental animals. Another research direction could be the study of oxidative stress imbalance caused by dust exposure as a possible reason for impaired germ cell formation.

## 5. Conclusions

This study demonstrated a significant influence of Aral Sea salt aerosols on the histomorphometry of seminiferous tubules, which can lead to reproductive dysfunction and impaired spermatogenesis. Such disruptions may also be observed in men living in areas affected by dust storms, negatively influencing their reproductive health. The findings of this study can be utilized in planning measures to improve the demographic situation in the Aral Sea region of Kazakhstan. However, additional research is necessary to clarify the mechanisms by which dust–salt aerosols, originating from the dried-up bottom of the Aral Sea, affect spermatogenesis.

## Figures and Tables

**Table 1:** Morphometric characteristics of rat testicular tissues under different durations of exposure to Aral Sea salt aerosols.

Morphometric Characteristics	Control	Experimental Groups				
		1	2	3	4	5
Diameter of convoluted seminiferous tubules, µm ^1^ H = 154.4 *p* < 0.001	293.5 (276.1; 305.7)	194.0 (179.3; 205.6) * p(c) < 0.001	216.3 (207.3; 231.7) * p(c) < 0.001 *p*(1–2) = 0.269	189.7 (178.0; 201.8) * p(c) < 0.001 *p*(1–3) = 1 *p*(2–3) < 0.001	286.6 (264.0; 303.6) *p*(c) = 1 *p*(1–4) < 0.001 *p*(2–4) < 0.001 *p*(3–4) < 0.001	285.0 (266.3; 301.7) *p*(c) = 1 *p*(1–5) < 0.001 *p*(2–5) < 0.001 *p*(3–5) < 0.001 *p*(4–5) = 1
Thickness of spermatogenic layer, µm ^1^ H = 75.87 *p* < 0.001	82.3 (76.6; 87.0)	64.1 (59.5; 68.1) * p(c) < 0.001	74.5 (69.7; 80.0) * p(c) = 0.047 *p*(1–2) = 0.002	67.1 (64.9; 69.8) * p(c) < 0.001 *p*(1–3) = 0.172 *p*(2–3) = 1	75.3 (69.7; 81.3) *p*(c) = 0.464 *p*(1–4) < 0.001 *p*(2–4) = 1 *p*(3–4) = 0.52	64.1 (56.0; 68.3) * p(c) < 0.001 *p*(1–5) = 1 *p*(2–5) < 0.001 *p*(3–5) = 0.059 *p*(4–5) < 0.001
Number of Sertoli cells per tubule ^1^ H = 29.36 *p* < 0.001	15.5 (12.0; 18.0)	8.0 (6.0; 10.0) * p(c) < 0.001	11.0 (8.8; 12.0) *p*(c) = 0.189 *p*(1–2) = 0.16	11.0 (7.8; 14.3) *p*(c) = 0.09 *p*(1–3) = 0.322 *p*(2–3) = 1	9.0 (5.0; 12.3) * p(c) = 0.001 *p*(1–4) = 1 *p*(2–4) = 1 *p*(3–4) = 1	10.0 (8.0; 14.0) * p(c) = 0.022 *p*(1–5) = 0.924 *p*(2–5) = 1 *p*(3–5) = 1 *p*(4–5) = 1
Vacuolization of Sertoli cells ^1^ H = 105.1 *p* < 0.001	1 (0; 1)	1 (0; 3) * p(c) = 0.005	1.5 (0; 3) * p(c) = 0.003 *p*(1–2) = 1	11.0 (8; 14) * p(c) < 0.001 *p*(1–3) < 0.001 *p*(2–3) < 0.001	2 (1; 3) * p(c) < 0.001 *p*(1–4) = 1 *p*(2–4) = 1 *p*(3–4) < 0.001	2 (0; 2) * p(c) = 0.005 *p*(1–5) = 1 *p*(2–5) = 1 *p*(3–5) < 0.001 *p*(4–5) = 1
Number of Leydig cells ^1^ H = 18.47 *p* = 0.002	17.0 (15.0; 19.3)	14.5 (11.0; 18.0) *p*(c) = 0.861	14.0 (12.0; 17.3) *p*(c) = 0.788 *p*(1–2) = 1	12.0 (10.8; 15.0) *p*(c) = 0.066 *p*(1–3) = 1 *p*(2–3) = 1	11.5 (10.0; 15.0) * p(c) = 0.034 *p*(1–4) = 1 *p*(2–4) = 1 *p*(3–4) = 1	10.0 (9.0; 12.1) * p(c) = 0.001 *p*(1–5) = 0.581 *p*(2–5) = 0.637 *p*(3–5) = 1 *p*(4–5) = 1
Johnsen score ^2^ H = 19.91 *p* = 0.001	9.16 ± 0.07	8.87 ± 0.11 *p*(c) = 1	8.74 ± 0.12 *p*(c) = 0.229 *p*(1–2) = 1	8.54 ± 0.11 * p(c) = 0.001 *p*(1–3) = 0.32 *p*(2–3) = 1	8.71 ± 0.11 * p(c) = 0.046 *p*(1–4) = 1 *p*(2–4) = 1 *p*(3–4) = 1	8.66 ± 0.12 * p(c) = 0.023 *p*(1–5) = 1 *p*(2–5) = 1 *p*(3–5) = 1 *p*(4–5) = 1

^1^ values are presented as median (Q1–Q3); ^2^ values are presented as mean ± Standard Error of the Mean (SEM); *—statistically significant differences from the control group (*p* < 0.05); *p*(c)—significance of the difference between control and experimental group; *p*(x–y)—significance of the difference between experimental group number x and experimental group number y.

## Data Availability

The original contributions presented in this study are included in the article. Further inquiries can be directed to the corresponding author.
